# A novel brain-computer interface based on audio-assisted visual evoked EEG and spatial-temporal attention CNN

**DOI:** 10.3389/fnbot.2022.995552

**Published:** 2022-09-30

**Authors:** Guijun Chen, Xueying Zhang, Jing Zhang, Fenglian Li, Shufei Duan

**Affiliations:** College of Information and Computer, Taiyuan University of Technology, Taiyuan, China

**Keywords:** brain-computer interface, audio-assisted visual evoked EEG, space division multiple access, spatial-temporal attention, convolutional neural network

## Abstract

**Objective:**

Brain-computer interface (BCI) can translate intentions directly into instructions and greatly improve the interaction experience for disabled people or some specific interactive applications. To improve the efficiency of BCI, the objective of this study is to explore the feasibility of an audio-assisted visual BCI speller and a deep learning-based single-trial event related potentials (ERP) decoding strategy.

**Approach:**

In this study, a two-stage BCI speller combining the motion-onset visual evoked potential (mVEP) and semantically congruent audio evoked ERP was designed to output the target characters. In the first stage, the different group of characters were presented in the different locations of visual field simultaneously and the stimuli were coded to the mVEP based on a new space division multiple access scheme. And then, the target character can be output based on the audio-assisted mVEP in the second stage. Meanwhile, a spatial-temporal attention-based convolutional neural network (STA-CNN) was proposed to recognize the single-trial ERP components. The CNN can learn 2-dimentional features including the spatial information of different activated channels and time dependence among ERP components. In addition, the STA mechanism can enhance the discriminative event-related features by adaptively learning probability weights.

**Main results:**

The performance of the proposed two-stage audio-assisted visual BCI paradigm and STA-CNN model was evaluated using the Electroencephalogram (EEG) recorded from 10 subjects. The average classification accuracy of proposed STA-CNN can reach 59.6 and 77.7% for the first and second stages, which were always significantly higher than those of the comparison methods (*p* < 0.05).

**Significance:**

The proposed two-stage audio-assisted visual paradigm showed a great potential to be used to BCI speller. Moreover, through the analysis of the attention weights from time sequence and spatial topographies, it was proved that STA-CNN could effectively extract interpretable spatiotemporal EEG features.

## Introduction

As an emerging human-computer interaction technique, the brain-computer interface (BCI) can realize the communication between the brain and the external devices without depending on the peripheral nervous and muscular tissues. The BCI can significantly improve the interaction experience for disabled people or some specific interactive applications including medical rehabilitation, healthcare, intelligent control, entertainment and so on (Chaudhary et al., 2016; Song et al., 2020). The scalp Electroencephalogram (EEG)-based BCI system has received more attention due to its easily used, relatively inexpensive, and high time resolution. Currently, some kinds of EEG signals with intentions modulated from the large neuronal activity are widely used in BCI systems including sensorimotor rhythm (SMR), steady-state visual evoked potential (SSVEP), and event-related potential (ERP). The SMR-based BCI usually requires a relatively long training time and even becomes ineffective after a certain amount of training for some users (Blankertz et al., 2010). The SSVEP-based BCI usually has a strong visual stimulation, which could cause the user's visual fatigue (Allison et al., 2014).

In the past few years, ERP-based BCIs have been widely investigated. One is the P300 speller, where a P300 component is elicited when the target character in a matrix is flashed with a small probability (Aloise et al., 2012). The P300 is a positive peak potential with a latency of about 300ms after the stimulus onset. To avoid flashing stimuli, the motion-onset visual evoked potential (mVEP) has been widely applied in BCI by attending to the target with a moving bar in an overt or covert way (Hong et al., 2009; Schaeff et al., 2012). The mVEP is composed of three main ERP components: P1 (P100), N2 (N200) and P2 (P200). The positive peak P1 with a latency of about 130 ms and the late negative peak N2 with a latency of 160–200 ms are considered as the main motion specific components (Zhang et al., 2015).

However, most of the ERP-based BCI must take a long time to output a target, where the stimuli must traverse all the target and nontarget with mutiple different time slices. To improve the detection speed, the dual-directional motion encoding paradigm was presented to reduce the stimuli presentation time by half (Liu et al., 2021). A new speller based on miniature asymmetric visual evoked potentials and space-code division multiple access (SDMA) scheme was developed, which can reduce stimuli time to implement an efficient BCI (Xu et al., 2018). For the SDMA scheme, the stimuli of targets and nontargets appear at different locations in the visual field simultanously, where an intended stimulus is attended to output the target quickly (Gao et al., 2014). Therefore, this study explored a new SDMA scheme to develop an efficient mVEP-based speller.

Compared with spontaneous EEG, the amplitude of single-trial ERP is so small that it is difficult to identify the target. Generally, to improve the signal-to-noise ratio (SNR) of ERP, averaging the EEG over several trials is used to obtain the discriminated ERP components. Nevertheless, it would decrease the output speed of the BCI system. An audiovisual hybrid BCI was designed to evoke stronger P100, N200, and P300 responses than the visual modality (Wang et al., 2015). The observed audiovisual integration effects can enhance the discriminability between target and nontarget brain responses. Moreover, an audiovisual P300-speller paradigm was proposed, which significantly improved the classification accuracies compared with the visual-based P300-speller (Lu et al., 2019). So, to enhance the quality of the ERP components, a semantically congruent audio-assisted mVEP paradigm was further used to output the target character in this study.

In addition, it is essential to decoding the ERP from a single-trial EEG to achieve fast and accurate target output. In some methods, the ERP components and spontaneous EEG were separated from a single-trial EEG based on a priori ERP pattern using wavelet transform (WT) (Quiroga, 2005), independent component analysis (ICA) (Lee et al., 2016) and so on. An iterative principal component analysis (PCA) method was proposed to extract single-trial ERP by reconstructing the principal components with a higher correlation with the target ERP (Mowla et al., 2016). Other methods aimed to improve the classification performance of single-trial ERP. The linear discriminant analysis (LDA) usually worked well for single-trial ERP classification. However, an accurate covariance matrix estimation was required in high-dimensional feature spaces. A shrinkage LDA was proposed to achieve excellent results for single-trial ERP classification (Blankertz et al., 2011). Meanwhile, a spatial-temporal discriminant analysis (STDA) algorithm was introduced to learn spatial and temporal projection matrices collaboratively by adopting matrix features, and the ill-conditional problem of covariance matrix can be effectively solved (Zhang et al., 2013). To enhance the SNR of ERP and classification accuracy simultaneously, current detection methods of single-trial ERP were reviewed, and the best performance of the xDAWN-based spatial filter and Bayesian LDA method was proved during a rapid serial visual presentation task (Cecotti and Ries, 2017). A data-adaptive spatiotemporal filtering method based on the clustering and array WT was proposed to improve the discriminative features of single-trial ERP (Molla et al., 2018). To adapt to the ERP diversities, the discriminative canonical pattern matching (DCPM) was proposed and obtained outperformed classification performance for the single-trial classification of EEG datasets including P300, mVEP components and so on (Xiao et al., 2020).

Recently, deep learning has been demonstrated that it can deal with EEG feature learning and classification effectively (Amin et al., 2019). A convolutional neural network (CNN) with a layer dedicated to spatial filtering was proposed to detect the single-trial ERP (Cecotti et al., 2014). The EEGNet, using the depthwise and separable CNN, was introduced to construct an EEG-specific model, which achieved comparably high performance for within-subject and cross-subject classification (Lawhern et al., 2018). Furthermore, a novel CNN model was proposed to better use the phase-locked characteristic to extract spatiotemporal features for single-trial ERP classification (Zang et al., 2021). However, due to the inter-trial and inter-subject variability of single-trial ERP, it is still challenging to build an efficient decoding strategy for single-trial ERP. Current studies have suggested that large inter-trial and inter-subject differences exist in the amplitude and latency of ERP components. So, it becomes crucial to construct an adaptive learning model to extract the spatial-temporal features from single-trial EEG.

In sum, there are still some current challenges to the application of the EEG-based BCI, including the friendly cognitive load and EEG characteristics-guided BCI classification algorithms (Xu et al., 2021). Compared with the flashing or flickering visual BCIs, the mVEP is a convenient way to encode targets with briefly moving stimuli (Libert et al., 2022b). On single trial classification, CNN can achieve comparable performance to both the LDA and support vector machine, but slightly less stable and interpretable (Vareka, 2020). In this study, similar to the Hex-o-Spell (Treder and Blankertz, 2010), a two-stage overt attention BCI speller combining with the mVEP and semantically congruent audio evoked ERP was designed to output a target by taking advantage of audiovisual properties. The main contributions of this paper are as follows.

(1) In the first stage, the different character groups coded with mVEP were presented simultaneously in the different locations of the visual field based on a new SDMA scheme to improve the efficiency of visual stimuli presentation.
(2) The target character was selected based on the audio-assisted mVEP in the second stage, which can enhance the quality of the ERP components.
(3) The spatial-temporal attention-based CNN (STA-CNN) was proposed to deal with single-trial ERP components learning and classification. The STA-CNN can effectively extract interpretable spatiotemporal EEG features by adaptively learning probability weights.

The rest of the paper is organized as follows: materials and methods are demonstrated in Section Materials and methods. Then experiment results of our proposed BCI speller are presented in Section Experiment results.. Finally, the discussion and conclusion of this paper are provided in Section Discussion and conclusions.

## Materials and methods

### Two-stage audio-assisted visual BCI paradigm

**Figure 1:**
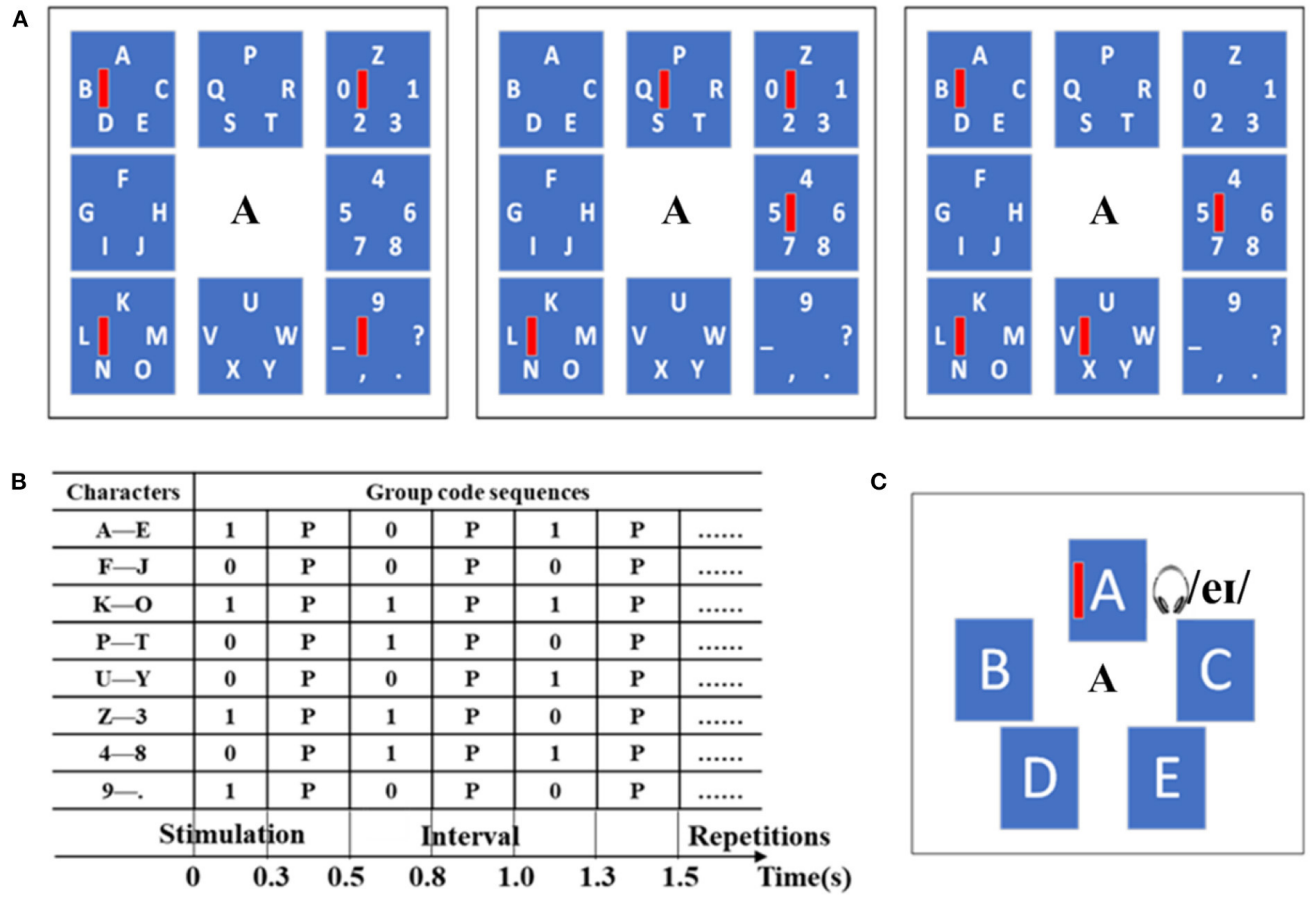
Speller paradigm example. **(A)** The first stage paradigm includes 3 sub-trials motion-onset stimuli based on the SDMA scheme. **(B)** A list of group code sequences for the first stage. **(C)** One example of the second stage paradigm includes motion-onset stimuli and pronunciation of character A simultaneously.

This study implemented a two-stage audio-assisted visual copy-spelling BCI, as shown in Figure 1. The paradigm was designed by using the Psychtoolbox in the Matlab 2012b environment. The visual stimuli were presented on a 17-inch LCD monitor with a 60 Hz refresh rate and 1440 × 900 pixels resolution. The audio stimuli were played by the headphone at a sensible volume.

In the first stage, forty alphanumeric characters were divided into 8 groups with 5 characters in each group, as shown in Figure 1A, wherein the size of each character group area was 280 × 280 pixels, and the size of each motion visual stimulus (red vertical bar) was 10 × 80 pixels. The target character group was selected based on the mVEP with a new SDMA scheme. For the SDMA scheme, three sub-trial motion visual stimuli sequences constituted eight parallel spatial channels. In each group, the red vertical bar appeared on the left side and moved rightward until it reached the right side, which lasted for 0.3 s as a brief motion-onset stimulus. Specifically, the motion-onset stimulus from left to right was regarded as code “1”, while no motion-onset stimulus was regarded as code “0”. The interval between two successive motion-onset stimuli was 0.2 s, and a complete stimulation sequence lasted for 1.5 s. Eight groups of code sequences were allocated to different character groups, as shown in Figure 1B. Take character A as an example, and its group code is “101”. That is, the ‘moving bar—none—moving bar' was presented by turns in the location of the top left group. During the spelling period, the motion-onset stimuli would be presented simultaneously for all character groups with different code sequences. The spatial information is embedded in the group codes. After three sub-trials of motion-onset stimuli in the first stage, the target character group would be selected.

Upon choosing of a character group, the speller switches to the second stage, and the target character can be selected based on the audio-assisted mVEP. One example of the second stage paradigm, including motion-onset stimuli and pronunciation of character A, is shown in Figure 1C. The motion-onset stimuli and semantically congruent audio (pronunciation) for each character would be presented simultaneously. During the presentation of audiovisual stimuli, when a moving bar was presented on a character, the pronunciation of the character was played by the headphone. Each group contained 5 characters, and the audiovisual stimuli of each character were presented randomly for 0.3 s with a time interval of 0.2 s. A complete stimulation sequence lasted for 2.5 s. Specifically, the audiovisual stimulation was similar to the “oddball” paradigm, and the target stimulation produced a P300 response. In the stimulation interface, the size of each character area was 170 × 250 pixels, and the size of each motion visual stimulus was 10 × 100 pixels.

### Subjects and experimental procedure

Ten healthy volunteers (22–26 years of age, 7 males, all right-handed) with normal hearing and normal or corrected to normal vision participated in this study. The experimental procedures were performed in accordance with the Declaration of Helsinki. The written informed consent was obtained from all subjects before the experiments, and the required tasks of the study were explained. After the experiments, the subjects received money for their participation. A total of 467 characters, including 10 sentences, were spelled in the copy-spelling task for each subject, with a 2 min rest between the sentences.

During the experiment, subjects were seated 50 centimeters in front of the LCD monitor. When a target character was introduced, it was shown on the screen center. In the first stage, the subjects were asked to pay attention to the center of the character group where the target character is located. In the second stage, the subjects were asked to pay attention to the target character. During the experiment, the subjects were asked to keep their heads as still as possible and blink less. And then, EEG was recorded using the Neuroscan SynAmps2 system with 64 channels referring to the international 10–20 electrode positions (Xu et al., 2018). The reference electrode was put in the position near Cz, and the ground electrode was put in the position near Fz. The impedance between the scalp and the electrode is <10 kΩ. The recorded EEG was bandpass-filtered at 0.1–100 Hz, sampled at a rate of 1000 Hz, and then stored in a computer.

After the EEG data were acquired, the recorded EEG data were re-referenced to the average of the bilateral mastoids (M1 and M2), filtered by a band-pass filter at 1–30 Hz, and down-sampled at 200 Hz. A 0.6 s time window was used to extract event-related data frames from – 0.1 to 0.5 s after stimulus onset, and 0.1 s baseline correction was applied in the first and the second stages. The format of a single trial EEG data in both two stages was a matrix of 62 channels × 100 time samples.

### Spatial-temporal attention CNN model

To enhance the discriminative event-related features from spatial-temporal domains, the spatial-temporal attention CNN (STA-CNN) model is proposed, which consists of four modules, as shown in Table 1.

**Table 1:** Parameters setting of STA-CNN model.

**Module**	**Layer**	**#Filters**	**Size**	**Strides**	**Output**	**Options**
1	Input				(62, 100)	
	Reshape				(1, 62, 100)	
	Conv2D	16	(1, 50)	1	(16, 62, 100)	Padding = (24, 25)
	BatchNorm				(16, 62, 100)	
	Activation				(16, 62, 100)	ELU
2	TemporalAttention				(16, 62, 100)	
	Conv2D	32	(1, 51)	1	(32, 62, 50)	
	BatchNorm				(32, 62, 50)	
	Activation				(32, 62, 50)	ELU
	Dropout				(32, 62, 50)	*P* = 0.5
3	ChannelAttention				(32, 62, 50)	
	Conv2D	4	(62, 1)	1	(4, 1, 50)	
	BatchNorm				(4, 1, 50)	
	Activation				(4, 1, 50)	ELU
	MaxPool2d		(1, 5)	5	(4, 1, 10)	
	Dropout				(4, 1, 10)	*P* = 0.5
4	Flatten				(40)	
	Dense				(2)	Softmax

The module 1 is mainly used for temporal filtering, which contains a reshape layer, a convolutional layer (Conv2D), and a batch normalization (BN) layer. The reshape layer transforms the EEG data into the input format of the Conv2D layer. And then, we perform a convolutional step in time sequence, and a 2D convolutional filter of size (1, 50) and stride 1 is used to output 16 feature maps containing the EEG data at different band-pass frequencies. The time length of the output is still 100 due to a 2D zero-padding of size (24, 25, 0, 0). In addition, the BN layer is performed before the activation function to avoid the distribution shift (Ioffe and Szegedy, 2015), and the exponential linear unit (ELU) activation function is used.

The module 2 performs the temporal features extraction, including a temporal attention layer, a Conv2D layer, and a BN layer. In the temporal attention layer, we adopt the adaptive event-related features learning, which can assign weights to different time samples based on importance. Suppose the feature maps *T*∈*R*^*Ns*×*Nf*×*Nc*×*Nt*^ from the module 1, we first apply a grand average pooling (GAP) for each time sample from different channels to obtain temporal-wise statistics $\bar{T}\in R^{Ns\times Nf\times 1\times Nt}$, where *N*_s_ is the batch size, *N*_f_ is the number of filters, Nc is the number of channels, *N*_t_ is the number of time points. The temporal attention mechanism adopts two fully-connected (FC) layers, including a dimensionality-reduction Linear layer 1 with tanh activation function and a dimensionality-increasing Linear layer 2, to reduce model complexity and improve generalizability. Thus, the temporal attention mechanism is expressed as follows.


(1) $$A_{t}=softmax\;\left(Linear2\;\left(\tanh\;\left(Linear1\;\left(\bar{T}\right)\right)\right)\right)$$

where the softmax function transforms the importance of time points to a probability distribution. Finally, we consider probability as the weight to recode the feature maps *T* at each time point. Thus, the attentive temporal feature can be represented as follows.


(2) $$\begin{array}{l} T_{a}=T•A_{t} \end{array}$$

The design of temporal attention on different periods utilizes relatively stable latency of event-related features for different channels. Then, we further perform a convolutional step in time sequence, and a 2D convolutional filter of size (1, 51) and stride 1 is used to output 32 feature maps. The time length of output becomes 50 to reduce the temporal dimension. And the BN layer is performed before the ELU activation function. To prevent over-fitting, we use the Dropout technique (Srivastava et al., 2014) and set the dropout probability to 0.5.

To further extract spatial information from the feature maps, the module 3 consists of a spatial attention layer, a Conv2D layer, and a BN layer. Similar to the temporal attention layer, the spatial attention layer assigns weights to different channels based on importance. Suppose the feature maps *S*∈*R*^*Ns*×*Nf*×*Nc*×*Nt*^ from the module 2, we apply a GAP for each channel of feature maps to obtain channel-wise statistics $S\in R^{Ns\times Nf\times Nc\times 1}$. The spatial attention mechanism also adopts two FC layers 3 and 4, which are expressed as follows.


(3) $$A_{s}=softmax\;\left(Linear4\;\left(\tanh\;\left(Linear3\;\left(\bar{S}\right)\right)\right)\right)$$

Finally, we consider probability as the weight to recode the feature maps *S* in each channel as follows.


(4) $$\begin{array}{l} S_{a}=S•A_{s} \end{array}$$

Compared with the traditional channel attention (Woo et al., 2018), this study only utilizes the average pooling instead of the sum of average and maximum pooling to become insensitive to the noise in EEG feature learning. Then, to learn a spatial filter, we further perform a 2D convolutional filter of size (62, 1) and stride 1 to output 4 feature maps. The BN layer is used before the ELU activation function. A maximum pooling layer of size (1, 5) and stride 5 is utilized to reduce the feature dimensions. To prevent over-fitting, we use the Dropout technique and set the dropout probability to 0.5.

In the module 4, after feature maps are flattened into vectors, a dense layer with the softmax function is used as the classifier of the model. The output size of the dense layer is set to 2, which corresponds to the target and non-target classes.

**Figure 2:**
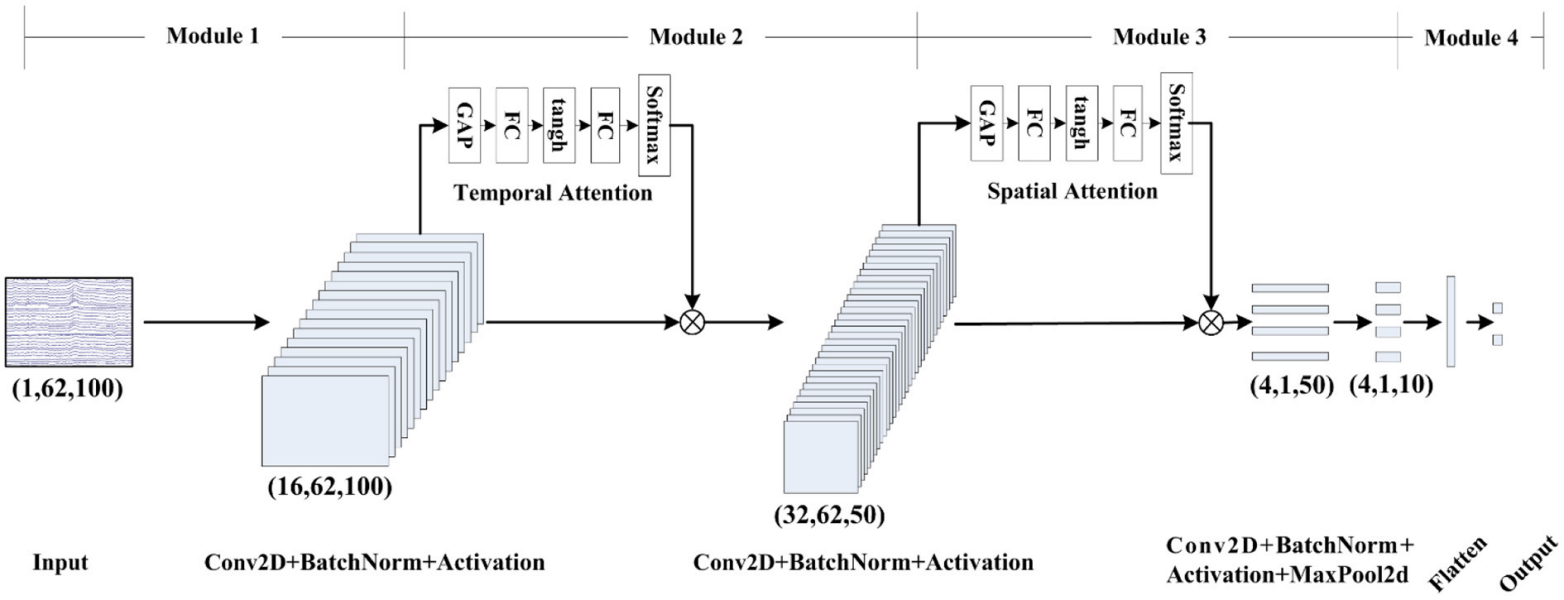
Schematic diagram of STA-CNN model.

In summary, we have designed a model, as shown in Figure 2 to extract spatial-temporal features and classification from single-trial EEG data. The model was trained using the Adam optimizer and the categorical cross-entropy loss function in PyTorch. We ran 300 training iterations and performed validation stopping, saving the model weights when we got the lowest loss of validation set.

## Experiment results

### ERP components analysis

The performance of the proposed two-stage audio-assisted visual BCI paradigm and the STA-CNN model was evaluated using the EEG recorded by our experiment in Section Materials and methods. A total of 467 characters, including 10 sentences, were spelled for each subject. Hence, 714 target EEG segments and 687 nontarget EEG segments in the first stage, and 467 target EEG segments and 1868 nontarget EEG segments in the second stage were recorded for each subject.

**Figure 3:**
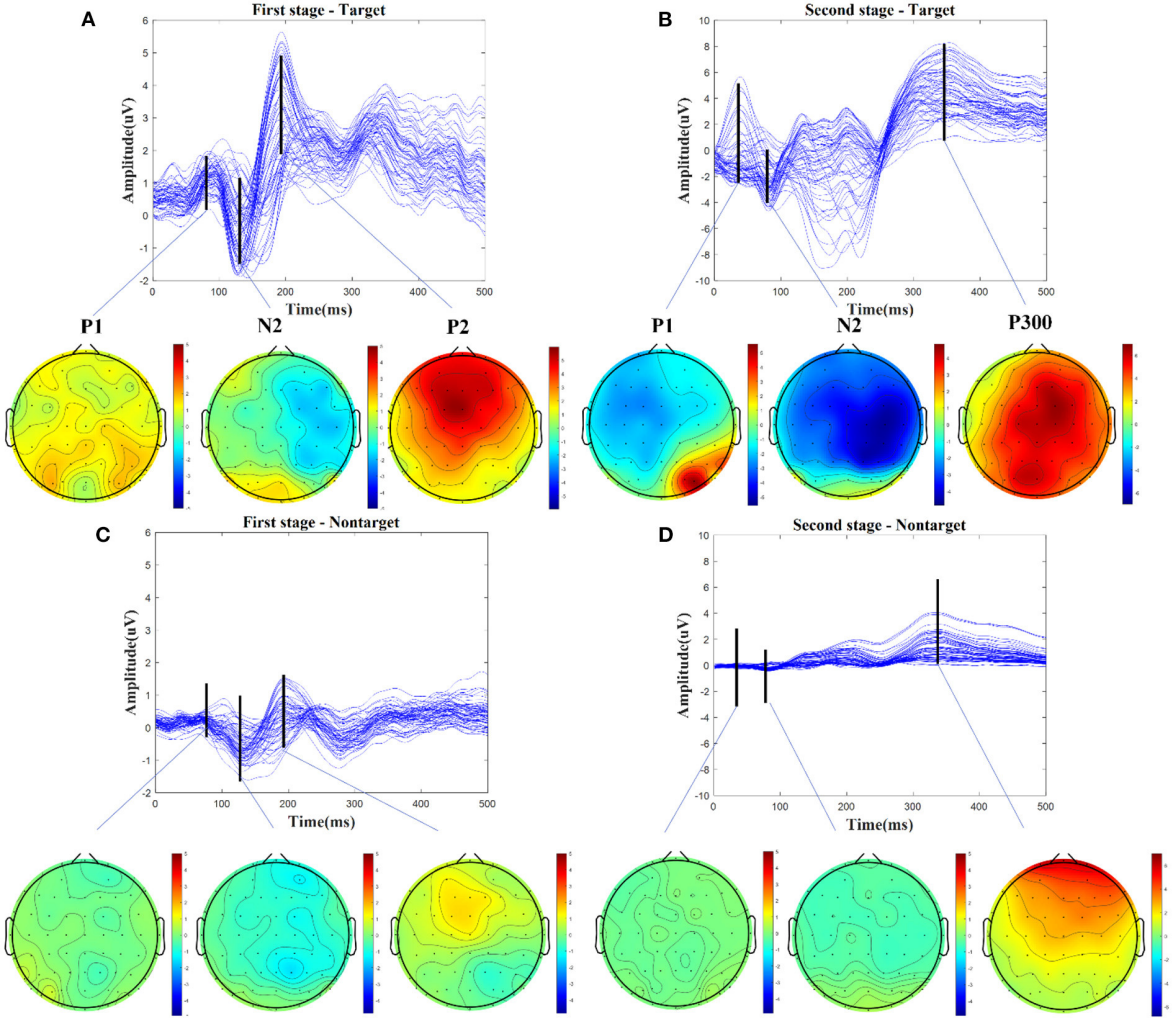
Averaged scalp potentials on 62 channels and topographies from the target and nontarget EEG epochs, for one example subject S10. **(A)** The mVEP components of P1, N2, and P2 and their topographies are in the first stage. **(B)** The audio-assisted visual evoked components of P1, N2, and P300 and their topographies are in the second stage. **(C)** The nontarget-related signal and topographies are in the first stage. **(D)** The nontarget-related signal and topographies are in the second stage.

We firstly analyzed the ERP components evoked from the audio-assisted visual BCI paradigm. The grand average of the target and nontarget EEG epochs in the first stages and the second stages for each subject were calculated separately. Figure 3 illustrates the averaged scalp potentials on 62 channels and topographies from the target and nontarget EEG epochs for one example subject S10. The amplitude of some time periods of target-related signal in Figures 3A,B is obviously higher than that of nontarget-related signal in Figures 3C,D. The three main time periods in Figure 3A with latencies of about 90, 130, and 195 ms after the motion-onset stimulus could be categorized as the P1, N2, and P2 components of mVEP. Compared with Figure 3A, the latencies of the mVEP components in Figure 3B become shorter, which are about 50, 90, and 180 ms after the motion-onset stimulus. This may be due to the earlier picked-up target cues in the first stage and the congruent audiovisual integration effect (Hessler et al., 2013; Simon and Wallace, 2018). Besides the mVEP components, there is an obviously larger amplitude with a latency of 350 ms in Figure 3B, which could be categorized to the P300 component evoked by the audio-assisted visual oddball paradigm. The above-mentioned P1, N2, P2, and P300 components are distributed in the central, temporo-occipital, and associate parietal cortical areas and dominate in the right hemisphere, which are consistent with the previous findings about mVEP and P300 components (Kuba et al., 2007; Guo et al., 2008; Belitski et al., 2011).

**Figure 4:**
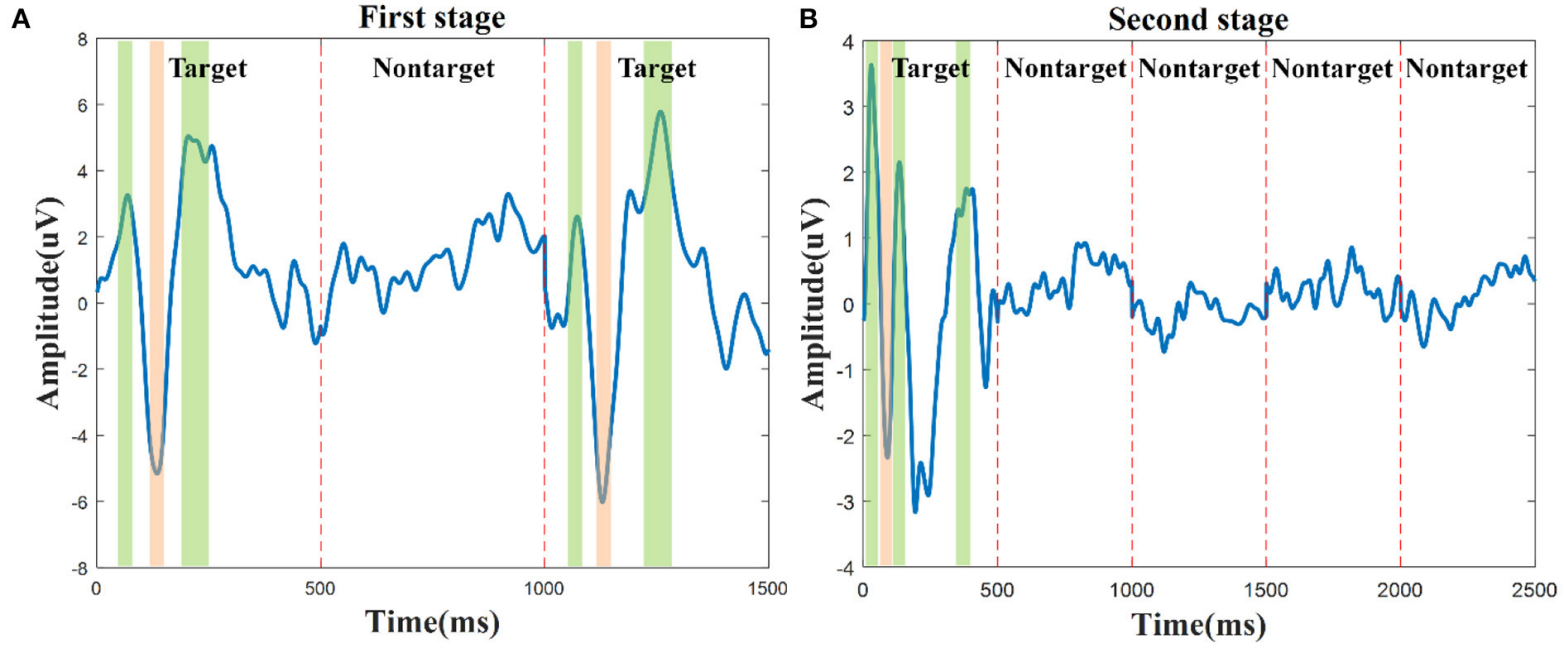
The grand average of target and nontarget signals during the character A spelling process for S10 was recorded on channel P4. **(A)** The mVEP of the group characters A-E in the first stage, P1, N2, and P2 are the three main target components. **(B)** The audio-assisted visual ERP components of character A in the second stage, P1, N2, P2, and P300 are the main target components.

Moreover, to further analyze the ERP components during the character spelling process, taking character A as an example, the grand average of target and nontarget related signals in the first stage and the second stage on channel P4 are shown in Figure 4. The reason for choosing channel P4 is based on the significant differences between target and nontarget related signals shown in Figure 3 and the previous findings (Zhang et al., 2015). In the first stage, the three epochs could be coded as “101” for the group code sequence of characters A-E. The amplitudes of the target-related mVEP components, including P1, N2, and P2 (color area) during the first and third epochs are significantly higher than that of the nontarget-related signal during the second epoch, as shown in Figure 4A, where the mVEP components from the first 500 ms and the third 500 ms epochs are coded as “1” and the nontarget signal in the middle 500 ms epoch is coded as ‘0'. In the second stage, besides the P1, N2, and P2 components, the audio-assisted visual target stimuli can evoke obviously P300, while the nontarget stimuli had no obvious ERP components. Therefore, the proposed two-stage audio-assisted visual stimulus paradigm shows great potential to be used for BCI speller.

### Single-trial ERP classification performance

Due to the different spatial-temporal characteristics of the ERP components in both two stages, the STA-CNN classification models were constructed based on the single trial EEG data in the first stage and the second stage, respectively. For the integrity of spelling characters, 448 target EEG segments and 452 nontarget EEG segments for 300 characters in the first stage were used as the training set for each subject, ten percent of which were used as the validation set. Similar to the first stage, 300 target EEG segments and 1200 nontarget EEG segments in the second stage were used as the training set, 10% of which were used as the validation set. For the training set, target samples were replicated 3 times to ensure the same number of samples from the two categories in the second stage, which could avoid model deviation caused by an unbalanced sample number. The remaining EEG segments for 167 characters were used as the test set in both stages. The classification performance was evaluated by the metrics: accuracy and F1-score.

In this part, the proposed STA-CNN was compared with several LDA variants and deep learning methods, including STLDA, DCPM, DeepLDA (Wu et al., 2017) and EEGNet, to validate the single-trial ERP classification performance. For the above comparison methods, the model parameters in this study were set by referring to the original literature. Tables 2, 3 present the classification results in terms of accuracy and F1-score for each subject using the above five methods. The classification accuracy of the second stage with the audio-assisted visual stimulus is higher than that of the first stage with visual stimulus. The overall classification results vary with different subjects, and subjects 2, 4, 7 and 10 could obtain higher spelling performance. The average classification accuracy of proposed STA-CNN across all subjects can reach 59.6% and 77.7% in the first and second stages, which are always higher than those of the comparison methods. The paired samples *t*-test was utilized to verify whether there were significant differences in classification performance between STA-CNN and other comparison methods. The results show that the STA-CNN can obtain significantly higher accuracy (*p* < 0.01) and F1-score (STLDA: *p* = 0.03 < 0.05, DCPM: *p* = 0.041 < 0.05, DeepLDA: *p* = 0.014 < 0.05, EEGNet: *p* = 0.143) in the first stage, while the STA-CNN can obtain significantly higher accuracy (*p* < 0.01) and F1-score (EEGNet: *p* = 0.027 < 0.05, others *p* < 0.01) in the second stage.

**Table 2:** The classification accuracy and F1-score of 10 subjects using five methods in the first stage (%).

**Subject**	**Accuracy**					**F1-score**				
	**STLDA**	**DCPM**	**DeepLDA**	**EEGNet**	**STA-CNN**	**STLDA**	**DCPM**	**DeepLDA**	**EEGNet**	**STA-CNN**
S1	56.3	56.7	54.9	57.9	59.1	60.7	60.6	55.2	61.6	59.7
S2	74.9	74.3	77.1	78.0	78.2	76.0	75.1	78.1	79.1	78.6
S3	50.9	51.5	53.3	53.3	54.3	53.2	54.4	54.5	55.9	59.9
S4	60.3	60.1	62.1	62.5	63.7	58.8	60.6	59.1	60.2	62.6
S5	50.3	50.5	51.5	53.3	56.5	51.0	49.3	50.3	52.1	56.4
S6	48.7	50.3	52.3	50.5	50.5	52.0	54.3	52.1	47.5	50.4
S7	58.7	58.3	60.1	60.5	62.7	62.8	61.5	62.3	64.8	60.3
S8	50.5	50.5	50.5	52.1	53.7	52.6	50.4	52.9	53.3	55.2
S9	50.5	52.1	51.7	54.1	54.5	51.9	54.6	53.8	56.6	59.0
S10	56.9	54.5	57.3	61.1	63.3	59.3	57.0	57.4	61.4	64.6
Mean ± Std	55.8 ± 7.8	55.9 ± 7.3	57.1 ± 8.0	58.3 ± 8.1	59.6 ± 8.0	57.8 ± 7.7	57.8 ± 7.4	57.6 ± 8.0	59.2 ± 8.7	60.7 ± 7.4

**Table 3:** The classification accuracy and F1-score of 10 subjects using five methods in the second stage (%).

**Subject**	**Accuracy**					**F1-score**				
	**STLDA**	**DCPM**	**DeepLDA**	**EEGNet**	**STA-CNN**	**STLDA**	**DCPM**	**DeepLDA**	**EEGNet**	**STA-CNN**
S1	52.7	54.0	63.5	67.9	71.4	28.8	28.1	36.6	30.6	34.2
S2	74.5	72.1	72.0	74.7	79.2	48.9	42.5	41.2	49.2	52.5
S3	54.3	58.6	64.6	60.1	63.0	30.8	32.7	33.0	34.8	34.1
S4	83.1	85.4	87.3	87.7	88.7	64.1	67.7	70.1	71.3	72.8
S5	76.5	74.6	77.8	80.0	83.0	49.2	47.3	48.8	52.2	55.9
S6	61.3	57.0	68.0	65.5	69.6	34.2	34.4	35.7	41.2	39.5
S7	83.2	84.4	84.4	86.6	87.9	65.4	67.2	64.5	68.0	71.2
S8	59.4	58.6	56.3	60.5	65.5	24.5	32.7	28.0	25.7	26.8
S9	71.7	73.1	78.4	78.0	79.9	43.5	46.3	51.1	49.7	52.5
S10	78.3	79.5	87.1	87.7	89.0	60.2	61.9	72.2	71.5	75.5
Mean ± Std	69.5 ± 11.6	69.7 ± 11.8	73.9 ± 10.8	74.9 ± 10.9	77.7 ± 9.8	45.0 ± 15.1	46.1 ± 14.9	48.1 ± 16.0	49.4 ± 16.7	51.5 ± 17.6

Furthermore, according to the trained STA-CNN models in the first and second stages, we provide the total classification accuracy of the above-mentioned test set (EEG segments for 167 characters) to evaluate the effectiveness of the paradigm and classification method. In the first stage, the group (during 3 sub-trials) with the group code corresponding classifier output was chosen, and in the second stage, the character (out of 5 characters) with the highest classifier output was chosen. The total classification accuracy of 10 subjects is listed in Table 4. Notice that the chance level is 1/40 = 2.5% for the two-stage spelling paradigm. The total classification accuracy varied with different subjects and ranged from 27.0 to 61.7%. Herein, the total classification accuracy is greatly affected by the first stage, and once the spelling error occurs in the first stage, it should be returned to the group selection during the actual spelling process.

**Table 4:** The total classification accuracy of 10 subjects for the ablation study (%).

**Subject**	**Total classification accuracy**			
	**CNN**	**TA-CNN**	**SA-CNN**	**STA-CNN**
S1	25.8	30.5	29.9	31.7
S2	55.1	58.7	56.3	61.7
S3	24.0	26.4	24.6	28.1
S4	50.3	55.7	53.3	56.9
S5	46.1	53.9	47.9	55.7
S6	32.9	30.5	24.6	29.9
S7	52.4	59.0	57.5	59.9
S8	22.8	25.2	24.6	27.0
S9	35.9	43.7	40.1	44.3
S10	53.9	55.1	59.3	56.3
Mean ± Std	39.9 ± 13.1	43.9 ± 14.2	41.8 ± 14.8	45.2 ± 14.5

Meanwhile, we provided the ablation study to validate the effectiveness of the spatial-temporal attention module in the STA-CNN method. The CNN is the baseline model that removes the spatial and temporal attention modules. The TA-CNN is the model that removes the spatial attention module from STA-CNN. The SA-CNN is the model that removes the temporal attention module from STA-CNN. The structures and parameters of all these three models were set according to the STA-CNN model in Table 1. As shown in Table 4, compared with CNN, TA-CNN and SA-CNN achieve better performance, which validates the effectiveness of the spatial and temporal attention modules. Combing with the spatial-temporal attention module, the STA-CNN is more effective than the TA-CNN and SA-CNN. The TA-CNN extract effective target ERP features based on the difference of special time periods between the target and nontarget EEG signals, which can obtain higher classification accuracy than that of SA-CNN. And then, the paired samples *t*-test was utilized to verify whether these methods had significant differences. The results show that the STA-CNN can obtain significantly higher accuracy than other comparison methods (CNN: *p* = 0.001 < 0.01, TA-CNN: *p* = 0.002 < 0.01, SA-CNN: *p* = 0.005 < 0.01).

### Influence of spatial-temporal attention

**Figure 5:**
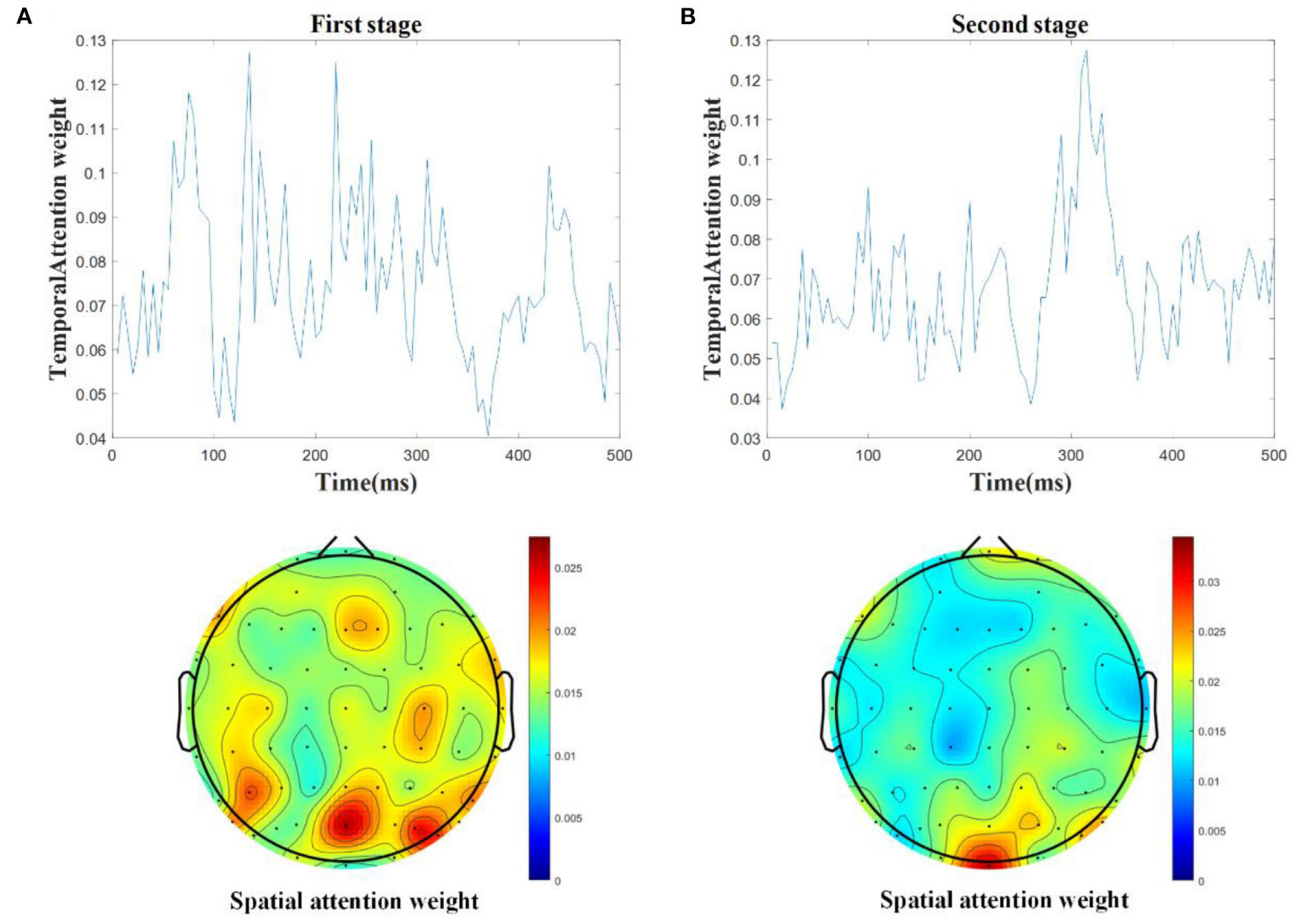
The average weights of temporal and spatial attention from the test samples based on STA-CNN using line charts and topographies in the first stage **(A)** and the second stage **(B)**.

The deep learning methods can automatically learn the EEG features, but it is difficult to determine if the spatial-temporal characteristics of ERP have been extracted efficiently. The spatial-temporal attention becomes essential to learn the individual spatial filters for particular time periods. In order to show the influence of spatial-temporal attention, Figure 5 shows the average weights of temporal and spatial attention from the test samples based on the STA-CNN model for the two stages.

In the first stage, as shown in Figure 5A, the results show that there are higher temporal weights nearby the time periods of 90, 130, and 210 ms, and higher spatial weights located at temporo-occipital cortical and parietal cortical areas. The time periods with higher temporal weights are similar to the latencies of mVEP in Figure 3A, and the higher spatial weights reflect the differences between target and non-target activated brain regions in Figures 3A,C. Similar to the first stage, as shown in Figure 5B, there are also higher temporal weights nearby the time periods of 50, 100, 180, and 310 ms, and higher spatial weights located at occipital and right temporo-parietal cortical areas in the second stage, which are similar to the latencies of ERPs and the differences between target and nontarget activated brain regions in Figures 3B,D. These results are consistent with the ERP components analysis in Section Introduction and Figure 3. The proposed STA-CNN benefits from spatial-temporal weights of attention mechanism to learn the ERP features effectively, and thus it can achieve superior performance.

## Discussion and conclusions

The mVEP-based paradigm is suitable for BCI speller application because it can encode the intentions as the identifiable target components and does not make subjects feel visual fatigue even for a long-time use (Liu et al., 2019). However, the problems restricting the practical application of mVEP-based BCI are the coding efficiency of the large command set and the decoding accuracy of the single-trial ERP due to low SNR (Lotte et al., 2018; Xu et al., 2020). Similar to telecommunication systems, the multiple targets coding strategy aims to simultaneously share the bandwidth from time, frequency, code and space with the least performance degradation (Gao et al., 2014). In this paper, the SDMA method was utilized to present multiple motion-onset visual stimuli in the different locations of the visual field simultaneously, as shown in Figure 1, which can effectively improve the coding efficiency of spelling intentions. For example, to achieve the presentation of 40 characters, at least 14 times presentations are required using a determinant matrix. In contrast, this paper requires 8 presentations, including 3 parallel mVEP stimuli for determining group codes and 5 audio-assisted visual stimuli for determining character codes. The purpose is to improve the SNR of ERP components by utilizing the integration effect of audiovisual stimuli. Suppose the group and character codes are presented in parallel based on mVEP, a target character can be coded with a maximum of 6 times presentation, which can achieve a higher output speed.

On the other hand, according to the characteristics of mVEP and P300 from the audiovisual stimulus, extracting temporal and spatial information from single-trial EEG is the key to effectively decoding target ERP (Wirth et al., 2020). The traditional method is the grand average to improve the SNR of ERP. The second one is to extract ERP components from a single EEG according to the prior knowledge, such as wavelet transform, PCA, ICA and so on, but the computational complexity is high, and the result is not good. The others are using classification algorithms to identify targets and nontargets by mapping the original EEG to the separable space, such as the LDA method and its variants for optimizing key temporal segments and spatial activation positions of ERP. The development of deep learning (Li et al., 2018) has obvious advantages for decoding ERP, especially EEGNet has achieved good results. Based on EEGNet, this paper further introduces a spatial-temporal attention mechanism, which can effectively learn the key spatial-temporal features and make the deep learning method better interpretable. As seen in Figure 5, the spatial-temporal attention mechanism can obtain larger weighted values in the time period corresponding to the active components of mVEP and P300, as well as in the spatial channels corresponding to the active brain areas of the target. Moreover, the deep learning method can realize end-to-end feature learning, thereby improving the adaptive ability between different subjects or trials.

The high reliability and robustness of audiovisual BCI should be furtherly considered for different subjects, different times, and different scenarios (Liu et al., 2020). According to the results in Table 4, the total classification accuracy of our paradigm is greatly affected by the first stage, which still needs to be improved. Due to the visual interference in the first stage, the classification accuracy of SDMA-based mVEP is not high. According to the literature (Lu et al., 2020), audiovisual integration could enhance the activation of attention-related brain areas. We tried to introduce the semantically congruent audio (pronunciation) to enhance the strength of the target ERPs in the second stage. The experimental results showed that the classification accuracy in the second stage was higher than in the first stage, which proves the audio-assisted effect's positive influence. But there are 5 characters that need to be traversed one by one in the second stage, which would lead to a decrease in presentation efficiency. To improve the efficiency of the BCI paradigm, we analyze further possible strategies, including novel paradigms to enhance the EEG features, such as the leftwards or rightwards motion-onset stimuli translating (Libert et al., 2022b) and the two-dimensional auditory stimuli with both pitch (high/medium/low) and direction (left/middle/right) (Hohne et al., 2011), and the stable classification algorithm of ERP for cross subjects or scenarios, such as the analytic beamformer transformation (Libert et al., 2022a), ternary classification method (Zhang et al., 2021) and some transfer learning methods.

This study proposed the spatial-temporal attention CNN method for decoding a novel audio-assisted mVEP-based BCI speller. A two-stage stimulation framework combined with mVEP and semantically congruent audio evoked P300 was designed based on a new SCDMA scheme to improve efficiency. Meanwhile, the STA-CNN method was proposed to deal with single-trial ERP components learning and classification. Specifically, the spatial-temporal attention mechanism can enhance the discriminative event-related features by adaptively learning probability weights. The experiment results, obtained from a dataset including 10 subjects, showed that the classification accuracy and F1-score were significantly improved using the proposed STA-CNN compared with the LDA variant and deep learning methods. Moreover, through the analysis of the attention weights from time sequence and spatial topographies, it was proved that STA-CNN could effectively extract interpretable spatiotemporal features. It is possible to extend the proposed strategy in the mVEP-based BCI system in the online test scenario, and future studies are needed to avoid the mutual interference of different intentions in the SDMA scheme and develop a robust classification algorithm of ERP.

## Data availability statement

The raw data supporting the conclusions of this article will be made available by the authors, without undue reservation.

## Ethics statement

The studies involving human participants were reviewed and approved by Taiyuan University of Technology. The patients/participants provided their written informed consent to participate in this study.

## Author contributions

GC and XZ contributed to the conception and design of the study. GC organized the database, performed the analysis, and wrote the first draft of the manuscript. JZ, FL, and SD contributed to the manuscript revision. All authors participated to the scientific discussion. All authors contributed to the article and approved the submitted version.

## Funding

This work was supported in part by the National Natural Science Foundation of China (Grant Nos. 62201377, 62271342, 62171307, and 12004275), Research Project Supported by Shanxi Scholarship Council of China (Grant No. 2022-072), Scientific and Technological Innovation Project in Higher Education Institutions of Shanxi Province, China (Grant No. 2019L0189), and MOE of PRC Industry-University Collaborative Education Program (Grant No. 202002035019, Kingfar-CES Human Factors and Ergonomics).

## Conflict of interest

The authors declare that the research was conducted in the absence of any commercial or financial relationships that could be construed as a potential conflict of interest.

## Publisher's note

All claims expressed in this article are solely those of the authors and do not necessarily represent those of their affiliated organizations, or those of the publisher, the editors and the reviewers. Any product that may be evaluated in this article, or claim that may be made by its manufacturer, is not guaranteed or endorsed by the publisher.
